# Elevated level of anterior gradient-2 in pancreatic juice from patients with pre-malignant pancreatic neoplasia

**DOI:** 10.1186/1476-4598-9-149

**Published:** 2010-06-15

**Authors:** Ru Chen, Sheng Pan, Xiaobo Duan, Brad H Nelson, Rob A Sahota, Sarah de Rham, Richard A Kozarek, Martin McIntosh, Teresa A Brentnall

**Affiliations:** 1 GI Division/Department of Medicine, University of Washington, Seattle, WA 98195 USA; 2 Department of Pathology, University of Washington, Seattle, WA 98195 USA; 3 BC Cancer Agency, Victoria, BC, V8R6V5 Canada; 4 Virginia Mason Medical center, Seattle, WA 98101 USA; 5 Division of Public Health, Fred Hutchinson Cancer Research Center, Seattle, WA 98109 USA

## Abstract

**Background:**

Pancreatic intraepithelial neoplasias (PanINs) are precursors of malignant pancreatic cancer, an ideal stage for early cancer detection. We applied quantitative proteomics to identify aberrantly elevated proteins in pancreatic juice samples derived from patients with PanIN3.

**Results:**

Twenty proteins were found elevated in all three PanIN juices by at least two-fold. Among these proteins, anterior gradient-2 (AGR2) was found to be 2-10 fold elevated in PanIN3 juice samples analyzed by quantitative proteomics. An ELISA assay was developed to evaluate AGR2 levels in 51 pancreatic juice samples and 23 serum samples from patients with pancreatic cancer, pre-malignant lesions (including PanIN3, PanIN2, Intraductal Papillary Mucinous Neoplasms (IPMNs)) and benign disease controls (including chronic pancreatitis). AGR2 levels in the pancreatic juice samples were found significantly elevated in patients with pre-malignant conditions (PanINs and IPMNs) as well as pancreatic cancer compared to control samples (p ≤ 0.03). By ROC analysis, the AGR2 ELISA achieved 67% sensitivity at 90% specificity in predicting PanIN3 juice samples from the benign disease controls.

**Conclusions:**

These results suggest that elevation of AGR2 levels in pancreatic juice occurs in early pancreatic cancer progression and could be further investigated as a potential candidate juice biomarker for early detection of pancreatic cancer.

## Introduction

Pancreatic cancer is the fourth leading cause of cancer death in the United States [1]. The poor prognosis could potentially be improved by development of biomarkers that could be used for early detection. Pancreatic intraepithelial neoplasia or PanIN, represents the precursor lesion for pancreatic ductal adenocarcinoma and is graded 1-3, with PanIN3 representing the stage just before cancer. Advanced PanIN lesions, e.g. PanIN3, would be an ideal stage to diagnose patients, as this represents a time point when intervention and cure is possible.

Pancreatic juice is a proximal body fluid and represents an opportune specimen for identifying biomarkers of pancreatic cancer. Cancer cells are preferentially shed into the ductal lumen, making juice a rich source of cancer associated markers. Previous studies have been conducted to investigate telomerase, microRNA, methylation, DNA mutation, and aberrant proteins as potential biomarkers in pancreatic juice from patients with pancreatic cancer or IPMNs (intraductal papillary mucinous neoplasms) [2-10]. However, to our knowledge, there has not been any study to evaluate pancreatic juice samples from patients with PanIN3 lesions.

In this study, we first applied mass spectrometry based quantitative proteomics to globally profile pancreatic juice samples from patients with histologically confirmed PanIN3 (referred as PanIN3 juice) to identify proteins with differential expression level in comparison to juice from benign disease controls. Among the differential proteins revealed, AGR2 was elevated in all PanIN3 juice samples analyzed. A recent study showed that AGR2 was highly expressed in the tissue of both PanIN lesions and pancreatic cancer [11]. The same study also demonstrated that AGR2 was secreted into culture media by pancreatic cancer cells. To further investigate AGR2 levels in pancreatic juice samples and sera, we developed AGR2-specific monoclonal antibodies (mAbs) and an ELISA to quantitatively detect AGR2 in pancreatic juice and blood samples. We compared the AGR2 levels in samples (juice and serum) from controls (including benign diseases and chronic pancreatitis), patients with pre-malignant lesions (PanIN2, PanIN3 and IMPNs), and patients with pancreatic cancer. Statistical analyses were used to determine significance between each sample group, and the sensitivity and specificity of AGR2 in separating cases from controls.

## Materials and methods

### Specimens

The specimens were collected in accordance with approved Human Subject's guidelines at the University of Washington and Virginia Mason Medical Center. The pancreatic juice samples were collected during endoscopic retrograde cholangiopancreaticography (ERCP) and immediately stored at -80°C. The control juice samples were from: 1) 7 cancer-free patients who were undergoing evaluation of Sphincter of Oddi dysfunction; 2) 11 patients who have benign pancreatic diseases, such as chronic pancreatitis. The PanIN2 juice samples were obtained from 6 patients who had histologically proven PanIN2 but without pancreatic cancer. The PanIN3 juice samples were obtained from 9 patients who had histologically proven PanIN3 but without pancreatic cancer. These patients with PanIN diagnoses had complete pancreas resection, and no cancer found in the pancreas[12]. The IPMN cases were benign to borderline without evidence of malignancy. The pancreatic cancer juice samples were from 8 patients who had pancreatic ductal adenocarcinoma including stages 2 to 4. The diagnosis of disease was made histologically or, in the case of the controls, by imaging in combination with supporting laboratory values. The patient demographics are presented in Table 1. Serum samples were obtained in red top tubes and processed within 4 hours using a uniform protocol. Once processed the serum was stored at -80°C and no more than 2 freeze thaw cycles were allowed for a specimen used in the ELISA studies. Six sera from patients with PanIN2-3 and 9 sera from pancreatic cancer patients were included in this study for AGR2 serum ELISA testing. The 9 healthy control sera were purchased from Innovative Research (Southfield, MI).

**Table 1:** Patient demographics in pancreatic juice

	Benign (N = 18)	Premalignant (N = 25)	Pancreatic cancer (N = 8)
Age			
Mean	47.75	54.88	69.5
Range	18-73	25-79	46-77
			
Sex			
Male	4	12	3
Female	14	13	3
Unknown	0	0	2
			
Benign			
Sphincter of Oddi	7		
Chronic pancreatitis	11		
			
Premalignant			
PanIN2		6	
PanIN3		9	
IPMN		10	
			
Malignant			
stage 2			1
stage 3			3
stage 4			2
stage undetermined			2

### Quantitative proteomics

The 4-plex iTRAQ (isobaric tags for relative and absolute quantification) method [13] was applied in combination with tandem mass spectrometry for quantitative profiling of the pancreatic juice proteome from PanIN3 cases and normal control. The control sample was generated by pooling equal volume of 5 pancreatic juice samples from patients with benign disease. Three hundred microliters of pancreatic juice samples from pooled normal juice, and three separate PanIN3 cases (a, b, and c) were subjected to iTRAQ labeling followed by mass spectrometer analysis using similar methods as previously described[14]. The MS/MS data were analyzed and processed using Trans-Proteomic Pipeline (TPP). All of the MS/MS spectra were searched against IPI human protein database (v3.38) using the SEQUEST algorithm[15]. The database search results were validated using the PeptideProphet [16] and ProteinProphet [17]. ProteinProphet probability score > 0.9 was used as the cut-off value for protein identification to ensure that the false positive rate (error rate) for protein identification was < 0.9%. iTRAQ quantification on peptide and protein abundance was achieved using LIBRA program[18]. More information about Trans-Proteomic Pipeline, PeptideProphet, ProteinProphet, LIBRA and other programs can be obtained from the Seattle Proteome Center http://tools.proteomecenter.org.

### Production of recombinant AGR2 protein and monoclonal antibodies

A cDNA encoding AGR2 protein (21-175 aa) was reverse transcribed from the OVCAR-3 cell line (ATCC, Manassas, VA) and verified by DNA sequence analysis. Recombinant AGR2 protein (rAGR2) was expressed in *Escherichia coli *with a GST tag and purified on GSTrap columns (GE Healthcare, Fairfield, CT) using the AKTA Prime (GE Healthcare) purification system. Full length, GST-tagged recombinant AGR3 (rAGR3) protein was purchased (Novus Biologicals, Littleton, CO). rAGR2 was used to immunize BALB/c mice, and spleens from mice with high-titer antibody responses to AGR2 were used to develop mAbs using standard hybridoma technology. Hybridomas were cloned by limiting dilution, and the supernatants from each hybridoma were screened against the GST-tagged immunogen and, as negative controls, other irrelevant GST-tagged recombinant proteins. mAb affinities for AGR2 were evaluated with a Biacore X100 instrument (GE Healthcare).

### AGR2 immunoprecipitation and mass spectrometric identification

To ensure that the newly developed AGR2.43 mAb was binding specifically to AGR2 protein, we performed an immunoprecipitation using this antibody and then sequenced the immunoprecipitated product using mass spectrometry. OV90 cell line lysate was used as the source of AGR2. OV90 cell line lysate (1 mg in 1 ml) was pre-cleared by incubation with 50 ul of Protein G resin (Pierce, Rockford, IL) for 30 minutes at 4°C with shaking. Protein G was pelleted by centrifugation and discarded. Five microgram of AGR2.43 antibody was coupled to 50 ul of Protein G resin by incubation for 1 hour at 4°C with shaking. The AGR2 antibody-Protein G complex was centrifuged, washed in PBS, centrifuged again and added to the pre-cleared OV90 cell line lysate and incubated for 2 hours at 4°C with shaking. The complex was washed and centrifuged 3 times and resuspended in PBS. The complex was dissociated from protein G by incubation with 50 ul of 100 mM glycine at pH 2.5. Following centrifugation and removal of the protein G pellet, the remaining sample was neutralized by addition of 1 M Tris-HCl pH 8.5 and further prepared as described below for Mass Spectrometry analysis.

The AGR2 immunoprecipitated pull-down product was buffer exchanged with 50 mM sodium bicarbonate, reduced with 20 mM dithiothreitol at 37°C for 1 hour and incubated with 20 mM iodoacetamide in the dark for 30 minutes to block the cysteine groups. The proteins were then digested with trypsin (Promega, Madison, WI) at a 1:50 ratio for 18 hours at 37°C. The obtained peptides were purified with a C18 column (The Nest Group, Southborough, MA) and subjected to LC MS/MS analysis using a linear ion trap mass spectrometer (LTQ, ThermoFinnigan, San Jose, CA). The resulting data was searched against IPI human protein database V. 3.38 for peptide and protein identification using SEQUEST algorithm. A 1% false positive rate based on ProteinProphet was used as a cut-off value for protein identification.

### Western Blotting

Cells from the human pancreatic cancer cell lines CFPAC-1 and MiaPaca were harvested and treated with lysis buffer (PBS; 0.05% TritonX-100) including protease inhibitor cocktail (Roche, Mannheim, Germany). Following sonication and centrifugation, cell lysate supernatants were quantified using a BCA quantification kit (Sigma, Oakville, ON, Canada) and stored at -80°C. Ten micrograms of cell line lysate and 10 nanograms of rAGR2 or rAGR3 were separated using NuPAGE Novex 4%-12% Bis-Tris gels at 200V for 40 minutes and transferred to nitrocellulose using the XCell SureLock Mini-Cell (Invitrogen) at 30V for 60 minutes. Membranes were blocked overnight in blocking buffer (50 mM Tris; 150 mM NaCl; 5% skim milk powder). Membranes were then incubated with 2 μg/ml AGR2.43 in blocking buffer with 0.1% Tween-20 for 4 hours, washed three times in TBST (50 mM Tris; 150 mM NaCl; 0.1% Tween-20), incubated for 1 hour in Goat anti-mouse IR-800 (LI-COR, Lincoln, NE), washed again and visualized with the Odyssey infrared imager (LI-COR). As a loading control, the same membrane was further probed with an anti-GAPDH antibody (STEMCELL Technologies, Vancouver, Canada), reprobed with Goat anti-mouse IR-800 and imaged on the Odyssey infrared imager.

### AGR2 ELISA

To develop the ELISA, antibody pairs were identified using hybridoma supernatants from AGR2 specific hybridoma clones with a Biacore X100 instrument (GE Healthcare). Mouse IgGs were purified using a protein G column (GE Healthcare) and labeled with biotin using a kit (Thermo Scientific). The mAb pair (mAbs AGR2.43 and AGR2.44) that showed the best sensitivity to specifically detect rAGR2 in both 1× Reagent Diluent (RD; R&D Systems, Minneapolis, MN) and in RD supplemented with 10% normal human serum (NHS) was selected. A sandwich ELISA was established using mAb AGR2.43 as capture, mAb AGR2.44 as detector and Superblock (Thermo Scientific) as blocking buffer. The ELISA was further optimized by determining the optimal incubation time and the concentration of the capture and detector antibody, the streptavidin-alkaline phosphatase conjugate (Applied Biosystems Inc, Foster City, CA) and the chemiluminescent CSPD^® ^Substrate to achieve the highest sensitivity.

To assess the performance characteristics of the AGR2 ELISA, we tested serially diluted rAGR2 protein (from 40 ng/ml to 0.055 ng/ml in RD or RD+10%NHS) and diluted human serum and cell line lysates (diluted 1/10 in RD) in triplicate in three independent ELISA experiments. For each experiment, a five-parameter log-logistic (5PL) standard curve was generated using EnVision software version 1.12 and GraphPad Prism, and used to back-calculate the concentration of the rAGR2 standards and AGR2 levels in the human serum samples and cell line lysates. The means, signal-to-noise (S:N) ratios, intra- and inter-assay standard deviations (SD), and percent coefficient of variances (%CV) were calculated.

AGR2 ELISAs were performed by researchers who were blinded to the case/control status of specimens. Costar white high binding 96 well plates (Corning, Corning, NY) were coated with 100 μl/well of 1.25 μg/ml purified mAb AGR2.43 in 0.1 M carbonate buffer (33.5 mM Na_2_CO_3_, 0.1 M NaHCO_3_, pH 9.6) and incubated overnight at 4°C. Plates were blocked with 200 μl/well of Superblock (Pierce, Rockford, IL) and incubated at room temperature (RT) for 2.5 hours. Plates were washed with a protocol including six wash steps in TBST using a Skanwasher plate washer (Molecular Devices, Union City, CA). Patient serum, control serum or pancreatic juice was diluted 1:10 in RD and incubated for 2 hours at RT on a shaker. All samples and controls were assayed in duplicate. Plates were washed and incubated with 100 μl per well of 0.25 μg/ml biotinylated mAb AGR2.44 in TBST for 2 hours at RT with shaking. Plates were washed and incubated with 100 μl per well streptavidin-alkaline phosphatase conjugate at 1:2500 in TBST for 1 hour on a shaker at RT. After washing, the plates were incubated with 100 μl/well of 0.4 mM chemiluminescent CSPD^® ^Substrate with Emerald-II™ Enhancer (Applied Biosystems) at RT for 20 min in the dark, read on an EnVision multilabel plate reader (PerkinElmer, Waltham, Massachusetts), and analyzed using Envision software 1.12.

### Immunohistochemistry (IHC)

Pancreatic tissues from 2 PanINs, 2 pancreatic cancers and 2 cancer free controls were analyzed by IHC. Pancreatic tissue blocks were sectioned at 5 μm onto slides and incubated for 30 minutes at 58°C. The slides were deparaffinized before placing in a Ventana Discovery XT autostainer (Ventana, Tucson, AZ) for immunohistochemical staining. Antigen retrieval was performed with a Ventana's standard CC1 protocol. The slides were first incubated with mAb AGR2.43 for 60 minutes, and the appropriate cross-adsorbed, biotinylated secondary antibody (Jackson Immunoresearch, West Grove, PA) was applied for 32 minutes. Bound antibodies were detected using the DABMap kit (Ventana), counterstained with hematoxylin (Ventana), and coverslipped manually with Cytoseal-60 (Richard Allan, Kalamazoo, MI). Two slides from each tissue block were stained in separate experiments. The specificity of mAb AGR2.43 was confirmed by blocking experiments. Specifically, incubation of mAb AGR2.43 with rAGR2, but not rAGR3, resulted in loss of signal in subsequent AGR2 IHC staining.

### Statistical analysis

For the purpose of statistical analysis, variables regarding patient diagnostics were grouped in the following manner: control (including all benign diseases from patients undergoing a work-up for Sphincter of Oddi dysfunction, and patients with pancreatitis), premalignant lesions (including PanIN2, PanIN3, and IPMNs), and malignant cancer groups (Table 1). Statistical analyses were performed using GraphPad Prism (La Jolla, CA). Differences in the AGR2 levels between groups of patients were tested for statistical significance using the Mann-Whitney test in case of two groups and the Kruskal Wallis test in case of more than two groups, respectively. Empirical receiver operating characteristic (ROC) curves were used to determine the sensitivity and specificity of AGR2 in separating cases from controls. ROC curve shows the trade-off between sensitivity and specificity as the threshold for defining a positive test is varied. Generalized linear models were used to test for association between the AGR2 values in serum and pancreatic juice. Statistical significance was defined as *P *< 0.05.

## Results

### Identification of elevated proteins in pancreatic juice samples from patients with PanIN3 by quantitative proteomics

**Figure 1:**
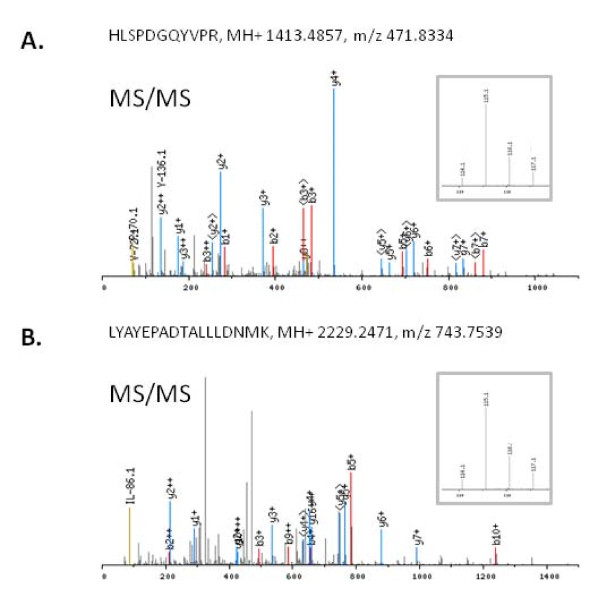
**Identification and quantification of AGR2 in pancreatic juice by iTRAQ labeling and tandem mass spectrometry**. Two unique peptides (HLSPDGQYVPR and LYAYEPADTALLLDNMK) from AGR2 were identified; the MS/MS spectra are presented in panel A and panel B respectively. The inserts are the iTRAQ reporting peaks at 114, 115, 116 and 117 representing relative intensity of the 4 samples compared: 114, pooled normal control juices; 115-117 were from PanIN case a, case b and case c respectively.

Quantitative proteomics analysis using stable isotope labeling (iTRAQ) and tandem mass spectrometry was applied to identify proteins abnormally elevated in the pancreatic juice samples from PanIN3 patients. A pool of 5 control pancreatic juice samples was used as a control for quantitative comparison. We identified twenty proteins displaying elevated levels by at least two-fold in all three PanIN3 juices when compared to normal control juice (Table 2). Among these elevated proteins, anterior gradient-2 (AGR2) was elevated by 10.47 ± 0.01; 3.96 ± 0.03; and 2.04 ± 0.01 folds in the three PanIN3 pancreatic juice samples respectively, comparing to the pooled control sample (Figure 1). A recent study using IHC showed that AGR2 was highly expressed in both PanINs and pancreatic cancer tissues and is secreted into the culture media of pancreatic cancer cell lines [11]. This, together with our results, prompted us to further investigated AGR2 for its utility as a biomarker for pancreatic cancer.

**Table 2:** Abnormally elevated proteins identified in PanIN3 juices by quantitative proteomics (by at least two-fold change)

gene symbol	protein description	ratio PanIN3 case a vs normal	PanIN3 case a SEM	ratio PanIN3 case b vs normal	PanIN3 case b SEM	ratio PanIN3 case c vs normal	PanIN3 case c SEM
AGR2	Anterior gradient-2	10.47	0.03	3.96	0.01	2.04	0.01
HIST1H2B	Histone H2B	34.78	0.04	8.53	0.03	10.01	0.01
LYZ	Lysozyme C	27.56	0.02	3.70	0.01	2.05	0.01
MUC5AC	Mucin-5AC	31.06	0.01	3.83	0.00	4.54	0.00
CA2	Carbonic anhydrase 2	9.58	0.02	3.18	0.01	1.97	0.01
KLK1	Kallikrein-1	3.25	0.06	3.12	0.08	11.52	0.02
ANXA5	Annexin A5	21.46	0.01	4.43	0.01	9.70	0.01
HIST2H4A	Histone H4	41.77	0.01	9.82	0.01	11.88	0.01
ANXA4	Annexin A4	18.39	0.02	4.28	0.01	5.11	0.01
ACTB	Actin, cytoplasmic 1, 2	17.98	0.01	6.13	0.01	4.41	0.01
SERPINA1	Alpha-1-antitrypsin	11.03	0.04	4.30	0.02	1.99	0.02
KRT8	Keratin, type II cytoskeletal 8	19.25	0.02	4.26	0.01	3.72	0.01
PRSS1	PRSS1 protein	4.56	0.01	15.35	0.02	30.46	0.03
YWHAE	14-3-3 protein epsilon	15.60	0.02	4.48	0.01	2.42	0.01
PPIA;LOC654188;PPIAL3	Peptidyl-prolyl cis-trans isomerase A	13.93	0.02	5.05	0.02	4.04	0.01
PSIP1	PC4 and SFRS1-interacting protein	2.68	0.01	17.91	0.00	12.16	0.01
AKR1B10	Aldo-keto reductase family 1 member B10	14.60	0.01	8.32	0.01	5.01	0.00
CLIC1	Chloride intracellular channel protein 1	5.84	0.04	3.22	0.04	4.08	0.04
C2	Complement C2	4.94	0.01	24.53	0.02	17.69	0.03
HNRNPA2B1	Heterogeneous nuclear ribonucleoproteins	7.13	0.04	2.64	0.01	2.51	0.04

### Production and evaluation of AGR2 antibodies

**Figure 2:**
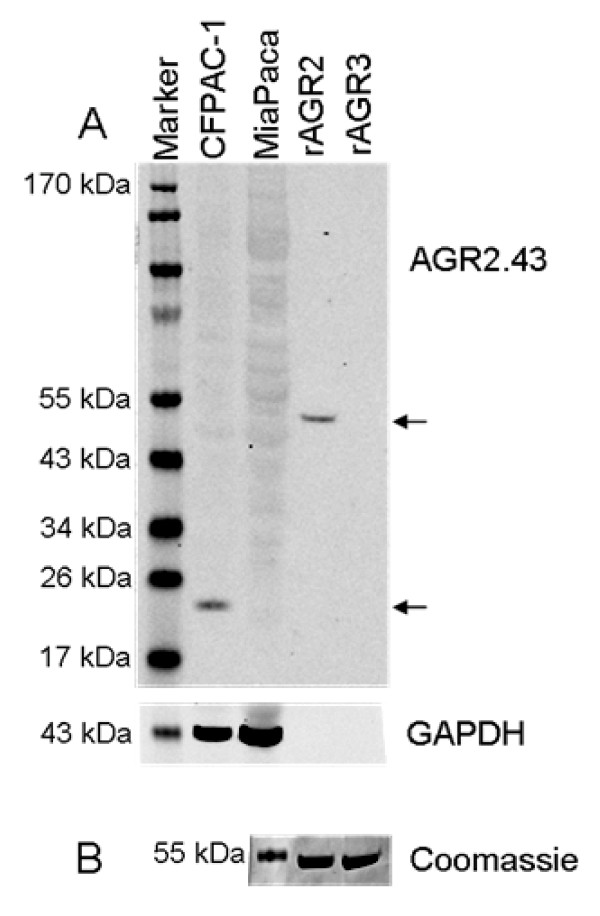
**Detection of AGR2 by Western blot with mAb AGR2.43**. A, Western blot of lysates from the human pancreatic cancer cell lines CFPAC-1 and MiaPaca, recombinant GST-tagged AGR2 (rAGR2) and AGR3 (rAGR3). As expected, mAb AGR2.43 detected the AGR2 protein (20 kDa) in CFPAC-1, but not MiaPaca lysates. The mAb also detected rAGR2 (45 kDa) but not rAGR3 (45 kDa). Note that rAGR2 and rAGR3 are of a higher molecular weight than their native counterparts because they carry a GST tag. B, Coomassie stained gel showing equivalent loading (1 μg each) of GST-tagged rAGR2 and rAGR3 proteins.

**Figure 3:**
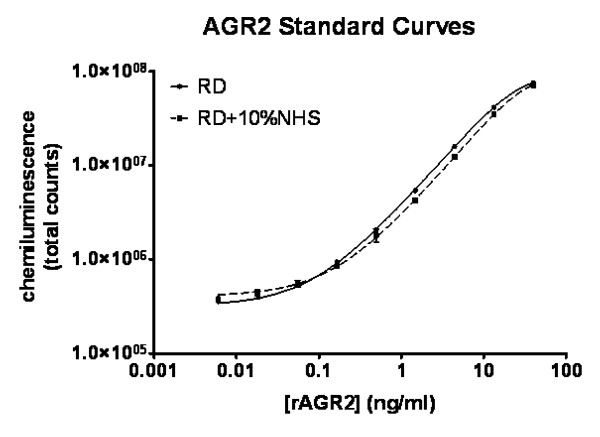
**AGR2 ELISA standard curves**. rAGR2 was diluted in either 1× Reagent Diluent (RD) or RD supplemented with 10% normal human serum (RD+10%NHS). A 3-fold dilution series of rAGR2 (from 40 ng/ml to 0.006 ng/ml) was generated in RD or RD+NHS. AGR2 ELISA was used to measure the signal for each sample in RD (closed circles) or RD+10%NHS (closed squares). Standard curves were generated for AGR2 in RD (solid line) or RD+10%NHS (dashed line) using the five-parameter log-logistic method (r^2 ^= 0.999 for both curves). The average of three replicates is presented. Error bars represent one standard deviation and are almost not apparent due to low intra-assay standard deviations.

**Figure 4:**
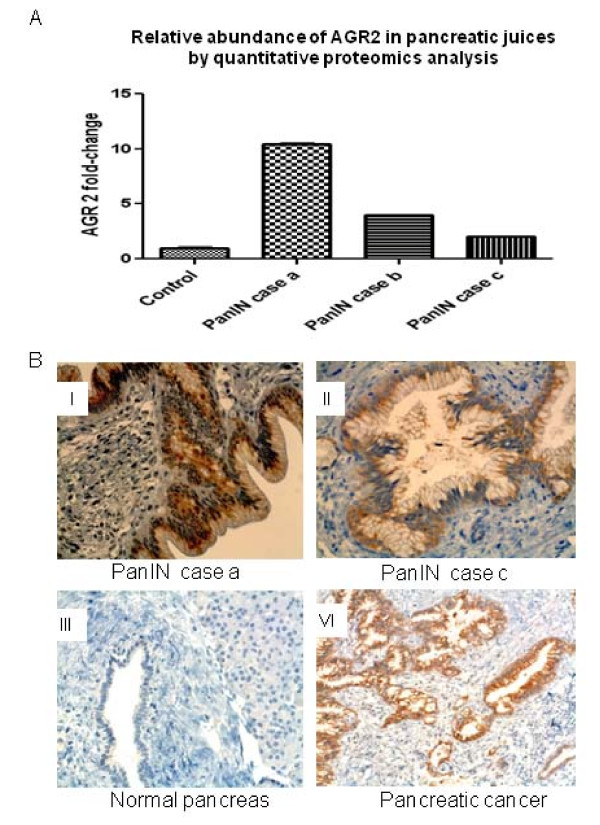
**Elevation of AGR2**. A. Fold-changes of AGR2 in pancreatic juice from patients with PanIN3 detected by quantitative proteomics analysis in comparison to controls. B. Detection of AGR2 by IHC in neoplastic pancreas ducts. Strong staining of AGR2 was detection in the neoplastic ducts of PanIN cases a and c (I and II), as well as the neoplastic region of pancreatic cancer (VI). AGR2 was not detected in the normal pancreas duct (III).

We generated multiple hybridoma clones producing antibodies specific for AGR2. Purified mAbs from these hybridomas were then assessed for their specificity to AGR2 using Western blotting, mass spectrometry, ELISA and IHC. By Western blot, mAb AGR2.43 detected the 45 kDa GST-tagged rAGR2, but not GST-tagged rAGR3 protein (Figure 2). This antibody also detected a 20 kDa protein corresponding to the predicted molecular weight of native AGR2 in the cell lysate of CFPAC-1, which expresses a high level of AGR2, but not in the pancreatic cancer cell line MiaPaca, which expresses a low level of AGR2 (Figure 2), nor in the normal pancreatic ductal cell line HPDE (data not shown). mAb AGR2.44 likely detects a discontinuous epitope as the antibody failed to detect the expected AGR2 band. mAb AGR2.43 was then used to immunoprecipitate proteins from the cell line OV90, and the immunoprecipitated proteins were analyzed by mass spectrometry. This revealed ten unique peptides covering 43% of the AGR2 protein; no peptides from the related protein AGR3 were detected (Table 3), suggesting no cross reactivity to AGR3. By ELISA, we could detect rAGR2 at concentrations as low as 0.055 ng/ml (Figure 3 and Table 4). In contrast, we failed to detect rAGR3 at concentrations of 4, 20, 100 or 500 ng/ml (data not shown). As expected, the ELISA detected higher levels of AGR2 protein in lysates from CFPAC-1 cells (120.7 ng/ml) compared to MiaPaca cells (14.6 ng/ml) (Table 4). By IHC, mAb AGR2.43 reacted with tissue sections from human PanINs and pancreatic cancer cases but not normal pancreatic epithelium (Figure 4B). Taken together, these data confirm that mAb AGR2.43 is specific to AGR2 and can be used for multiple applications.

**Table 3:** Peptides identified from the AGR2 pull-down product

AGR2 Peptide Observed	Sequence Position	Mass detected	PeptideProphet probability score
KDTKDSRPKLPQTLSR	51-66	1869.04	0.93
DSRPKLPQTLSR	55-66	1396.78	1.00
LPQTLSR	60-66	813.47	0.92
GWGDQLIWTQTYEEALYK	67-84	2200.05	1.00
GWGDQLIWTQTYEEALYKSK	67-86	2415.17	1.00
LAEQFVLLNLVYETTDKHLSPDGQYVPR	120-147	3244.68	1.00
NLVYETTDKHLSPDGQYVPR	128-147	2331.15	1.00
IMFVDPSLTVR	148-158	1276.68	1.00
IM[147]FVDPSLTVR	148-158	1293.83	1.00
ALKLLKTEL	187-195	1027.66	0.99

Note: [147] indicates oxidized methionine

**Table 4:** AGR2 ELISA performance characteristics

		Recombinant AGR2 Protein (ng/ml)							Human Serum	Panc. Cell Lysates	
		40	13.3	4.44	1.48	0.494	0.165	0.055		CFPAC1-1	MiaPaca
**All Assays**	**Mean (ng/ml)**	40.30	13.20	4.54	1.41	0.48	0.18	0.07	22.00	120.70	14.60
	**ratio (signal/noise)**	3559	1169	401	124	42	15	6.2	195	1065	129
											
**Intra-assay**	**SD (ng/ml)**	1.260	0.199	0.079	0.062	0.014	0.008	0.004	1.400	8.300	0.790
	**CV (%)**	3.1	1.5	1.8	4.4	2.9	4.6	5.9	6.2	6.8	5.4
											
**Inter-assay**	**SD (ng/ml)**	1.240	0.207	0.081	0.067	0.012	0.008	0.006	3.400	5.100	1.000
	**CV (%)**	3.1	1.6	1.8	4.8	2.6	4.5	9.1	16.0	4.3	6.8

SD: standard deviation; CV: coefficient of variation

### Performance characteristics of the AGR2 ELISA

We evaluated the AGR2 ELISA in different sample matrices using rAGR2, lysates from CFPAC-1 and MiaPaca cell lines, and a human serum sample. A 5PL standard curve was generated for each experiment and used to back-calculate the concentration of the rAGR2 standards and calculate AGR2 levels in lysates and serum. Figure 3 shows a standard curve of rAGR2 ranging from 0.006 ng/ml to 40 ng/ml. The curve produced an r-squared of 0.999 demonstrating a good fit to the 5PL curve. The limit of detection, calculated as the mean value for the blank controls plus two standard deviations was 0.024 ng/ml. The average signal to noise (S:N) ratios calculated from three separate experiments ranged from 3559 (40 ng/ml standard) to 6.2 (0.055 ng/ml standard) suggesting a limit of detection of at least 0.055 ng/ml AGR2 (Table 4). The intra- and inter-assay SD were calculated, and CVs ranged from 1.5%-5.9% and 1.6%-9.1%, respectively. CFPAC-1 and MiaPaca lysates and the human serum sample were determined to have 120.7 ng/ml, 14.6 ng/ml and 22.0 ng/ml AGR2 with intra-assay CVs of 6.8%, 5.4% and 6.2% respectively and inter-assay CVs of 4.3%, 6.8% and 16% respectively (Table 4). To assess the effects of serum on the AGR2 ELISA, we added 10% Normal Human Serum (NHS) to a subset of samples. The resulting standard curve produced an r-squared of 0.999 and closely mirrored the standard curve determined without serum (Figure 3).

### Correlation of AGR2 levels in pancreatic juice with its overexpression in neoplastic pancreas tissue

We next examined AGR2 expression by IHC in pancreatic tissues corresponding to the pancreatic juice samples from cases a and c in the above quantitative proteomics analysis. As shown in Figure 4B, AGR2 was highly expressed in the PanIN ducts from both PanIN cases a and c, and also highly expressed (strong staining) in pancreatic cancer, but not in normal pancreas epithelium (negative staining). Thus, the elevation of AGR2 level in the pancreatic juice was correlated with high expression in PanIN ducts. Previous study had reported high expression of AGR2 in neoplastic cells with 98% (56 of 57) positivity on pancreatic cancer and minimal staining in normal and pancreatitis tissues [11]. Our current study suggests that the detected AGR2 in pancreatic juices is likely originated from the pancreas.

### Significant elevation of AGR2 levels in the pancreatic juice samples from patients with pre-malignant pancreatic neoplasia

**Figure 5:**
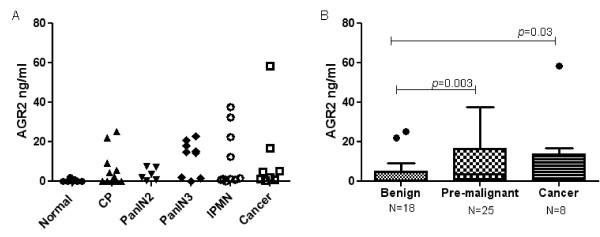
**A. AGR2 concentration in pancreatic juice as measured by ELISA**. CP: chronic pancreatitis. B. Boxplots of AGR2 concentration in pancreatic juices from patients with benign diseases (n = 20, including normal and chronic pancreatitis), pre-malignant lesions (n = 25, including PanIN2, PanIN3 and IMPNs), and pancreatic cancer (n = 8). Boxplot: the thin box marks out the 25th to 75th percentiles; the line marks the outer part of the distribution, and outside dots mark outliers.

We used ELISA to measure AGR2 levels in pancreatic juice samples from patients with benign pancreatic diseases (n = 18; including chronic pancreatitis), premalignant pancreatic neoplasia (n = 25; including PanIN2, PanIN3, and IPMNs), and pancreatic cancer (n = 8). As shown in Figure 5A and B, the level of AGR2 in pancreatic juice was significantly higher in patients with pre-malignant pancreatic diseases compared to benign disease controls (*p *= 0.003). The level of pancreatic juice AGR2 was also significantly higher in pancreatic cancer compared to controls (*p *= 0.03). The median pancreatic juice AGR2 level was not significantly different between males and females, different age groups, within subcategories of benign controls and premalignant pancreatic diseases (all p>0.05, Table 5).

**Table 5:** Median pancreatic juice AGR2 values by clinical-pathologic parameters

	number of case	Juice Median AGR2 (ng/ml)	*P*
Sex			0.35
Male	19	1.82	
Female	30	1.94	
			
Age			0.79
Y (<50)	24	2.09	
M (50-70)	17	1.82	
O (>70)	10	3.61	
			
Benign			0.12
Sphincter of Oddi	7	0.00	
Chronic pancreatitis	11	2.32	
			
Premalignant			0.36
PanIN2	6	2.88	
PanIN3	9	14.85	
IPMN	10	1.49	
			
Malignant	8	4.93	

**Figure 6:**
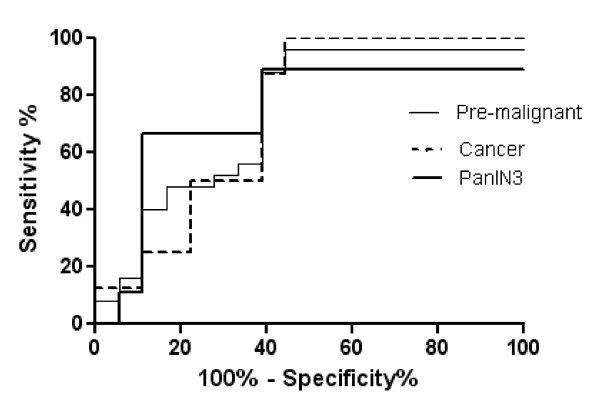
**ROC curves of AGR2 levels in pancreatic juice from patients with pre-malignant lesions, PanIN3 lesions, and pancreatic cancer compared to benign disease controls**.

ROC analysis was applied to evaluate the sensitivity and specificity of AGR2 in distinguishing premalignant and pancreatic cancer juice samples from benign controls (Figure 6). At 90% specificity, the AGR2 ELISA achieved 40% sensitivity in discriminating premalignant juice samples from the control group (Table 6). The area under curve (AUC) was 0.742. When we compared the ROC curve of the subset of PanIN3 juice samples to the control group, the AUC was slightly improved to 0.765. At 90% specificity, the AGR2 ELISA achieved 67% sensitivity in discriminating PanIN3 juice samples from the control group. Pancreatic cancer juice samples achieved a slightly lower AUC value (AUC = 0.729) and sensitivity than the premalignant group (Table 6). At 90% specificity, AGR2 had a sensitivity of 25% in discriminating pancreatic cancer juice samples from the control group.

**Table 6:** Pancreatic juice AGR2 ROC analysis

	Premalignant vs control	PanIN3 vs control	Cancer vs control
Area under curve (AUC)	0.742	0.765	0.729
Sensitivity at 95% specificity	16%	11%	11%
Sensitivity at 90% specificity	40%	67%	25%

### Non-correlation of AGR2 levels in pancreatic juice versus serum

**Figure 7:**
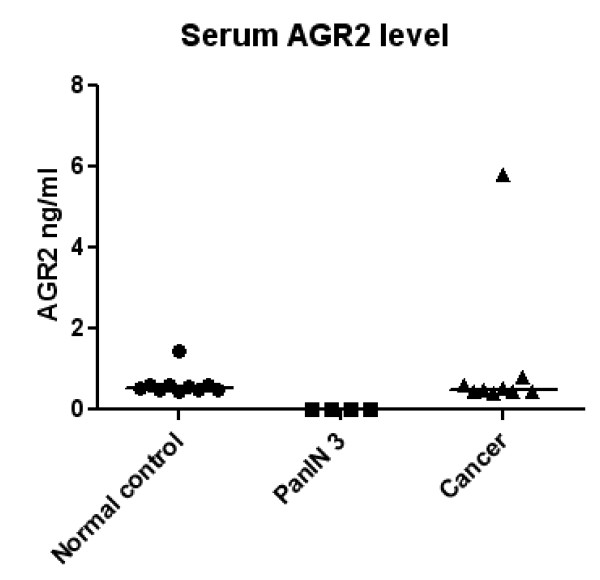
**Serum AGR2 levels detected by ELISA in normal controls (n = 9), PanIN3 (n = 4), and pancreatic cancer (n = 9)**.

AGR2 levels were measured by ELISA in six paired serum and juice samples (all from PanIN patients). AGR2 was not detectable in any of the six serum samples tested. Of the corresponding juice samples, five displayed positive AGR2 levels, ranging from 0.84 ng/ml to 17.91 ng/ml. Thus, AGR2 levels in pancreatic juice do not correlate with AGR2 levels in serum. Moreover, when we compared sera from pancreatic cancer cases and cancer-free controls, only 1/9 pancreatic cancer sera showed a high level of AGR2, compared to 0/9 cancer-free controls. Therefore, AGR2 levels were not significantly different between pancreatic cancer sera and sera from cancer-free controls (Figure 7, *p *= 0.27).

## Discussion

AGR2 is a secreted protein initially described in *Xenopus laevis *for being differentially expressed in neural development [19]. In *Xenopus*, the AGR2 homologue is critical in forming anterior structures during embryonic ectoderm development [20]. The function of AGR2 in human tissue is largely unknown, but overexpression of AGR2 has been reported in several cancers, including breast, prostate, lung and pancreas and in circulating tumor cells [11,21-25]. Recent evidence suggests that AGR2 can promote tumor growth, promote cancer cell survival, cell migration, and cellular transformation [11,19]. Detection of AGR2 in these previous studies was primarily performed on tissue or cell lines using RT-PCR, western blotting or IHC. One recent study showed that increased AGR2 in plasma is associated with ovarian cancer[26]. However, to the best of our knowledge, evaluation of AGR2 protein in plasma or other biological fluids has not been reported in pancreatic cancer.

In this study, we first used quantitative proteomics to identify elevated proteins in pancreatic juice. We found that AGR2 was elevated in all of the three pre-neoplastic pancreatic juice samples (PanIN3) analyzed by quantitative proteomics. The AGR2 levels in pancreatic juice correlated with its over-expression in matching pancreas tissues. These findings are in concert with previous studies that have also demonstrated increased expression of AGR2 in PanIN lesions [11,27] and pancreatic ductal adenocarcinoma tissues [11,23]. Along with these previous studies, it is evidenced that AGR2 is likely to be secreted into the pancreatic duct fluid early in pancreatic cancer progression. However, a recent study found that AGR2 is localized to the endoplasmic reticulum in intestinal epithelial cells and did not detect AGR2 in secretory granules or in the intestinal lumen[28]. Thus it is possible that only a fraction of AGR2 is actually secreted. And this could explain why pancreatic cancer patients do not have higher juice AGR2 level than the PanIN patients, while they have strong AGR2 staining in the cancer tissues. The mechanism governing AGR2 secretion is currently unknown.

To evaluate AGR2 levels in pancreatic juice and blood, we developed an ELISA assay to quantitatively measure AGR2 levels. We used mass spectrometry to verify that the monoclonal antibody used in the ELISA was indeed specific for AGR2. By ELISA, AGR2 levels in the pancreatic juice samples from patients with benign pancreatic disease, premalignant pancreatic neoplasia and pancreatic cancer were tested and compared. The pancreatic juice AGR2 level was significantly elevated in patients with premalignant lesions and pancreatic cancer compared to control samples. Further ROC analysis suggested that at 90% specificity, the AGR2 ELISA achieved 67% sensitivity in detecting PanIN3 juice samples from the control group. The ability to detect pancreatic cancer at an early stage could greatly benefit the patient management and improve the survival rate. Currently, it is only possible to detect PanIN3 histologically. Pancreatic juice CA19-9 levels could not separate patients with pancreatic cancer from chronic pancreatitis[29]. Moreover, CA19-9 is not elevated in the blood of PanIN3 patients, thus is not useful as a PanIN3 biomarker[12]. The discovery of AGR2 elevation in the pancreatic juice of PanIN3 patients in this study may provide a future opportunity to develop and test this candidate as juice biomarker for PanIN3.

While AGR2 levels were significantly elevated in the pancreatic juice from premalignant and pancreatic cancer patients, serum levels of AGR2 did not distinguish patients with premalignant or pancreatic cancer from control patients. This result supports the view that pancreatic juice, due to proximity to the tumor, can be a more sensitive and specific source for identifying biomarker candidates associated with pancreatic cancer or pre-cancer [30,31].

## Conclusion

In this study, we found that AGR2 was significantly elevated in the pancreatic juice from patients with pre-malignant conditions (PanINs and IPMNs) as well as pancreatic cancer compared to control pancreatic juice samples. AGR2 levels in pancreatic juice could potentially be used to aide in assessment of high-risk patients undergoing endoscopic procedures.

## Competing interests

The authors declare that they have no competing interests.

## Authors' contributions

RC and SP carried out the quantitative proteomics study. RC, SP, and XD drafted the manuscript. XD, BHN, RAS, SR generated monoclonal antibodies and carried out the immunoassays. RC, SP, BHN, RAK, MM and TAB participated in the design of the study and analysis of the data. All authors commented, read and approved the final manuscript.
